# Zinc Chelates from Low-Molecular-Weight Donkey-Hide Gelatin Peptides: Preparation, Characterization, and Evaluation of In Vitro Antioxidant Activity

**DOI:** 10.3390/foods14213671

**Published:** 2025-10-28

**Authors:** Wenxuan Han, Lili Yang, Yujie Fan, Yanyan Lv, Xiao Li, Yuhang Li, Siyu Li, Rong Liang

**Affiliations:** 1 Shandong Key Laboratory of Applied Technology for Protein and Peptide Drugs, School of Pharmaceutical Sciences and Food Engineering, Liaocheng University, Liaocheng 252059, China; m18053621746@163.com (W.H.); 13863540288@163.com (Y.L.);; 2 College of Food Science & Nutritional Engineering, China Agricultural University, Beijing 100083, China

**Keywords:** low-molecule-weight donkey-hide gelatin peptide, peptide–zinc chelates, preparation process optimization, structural characterization, antioxidant activity

## Abstract

This study synthesized a low-molecular-weight donkey-hide gelatin peptide–zinc chelate (LMW DHGP–Zn) using peptides derived from donkey-hide gelatin. Under optimized conditions (zinc concentration of 32 mg/mL, peptide-to-zinc mass ratio of 8:1, pH 6.5, 60 °C, 70 min), a maximum chelation rate of 41.34% ± 0.23% was achieved. Comprehensive characterization via scanning electron microscopy, ultraviolet–visible spectroscopy, fluorescence spectroscopy, zeta potential, particle size, Fourier transform infrared spectroscopy, and circular dichroism confirmed substantial structural and physicochemical alterations post-chelation. After chelation, the surface charge is neutralized, and the distribution of particles is more even. Furthermore, analysis indicated an elevated content of acidic amino acids (glutamic acid and aspartic acid), and spectroscopic data confirmed the coordination of zinc ions with amino and carboxyl groups on the peptide. Consequently, the LMW DHGP–Zn chelate demonstrated significantly enhanced ABTS radical cation scavenging activity. These results provide a scientific foundation for its potential application as a natural antioxidant in the food, cosmetic, and pharmaceutical industries.

## 1. Introduction

With growing health consciousness among consumers, the demand for functional foods has been steadily increasing. Novel functional food ingredients possessing bioactive properties, particularly antioxidant and anti-aging effects, have emerged as a prominent research focus in food science. Donkey-hide gelatin, a traditional Chinese medicinal and edible resource with a 2000-year history of application, contains various bioactive components including proteins [1], amino acids, trace elements, polysaccharides, chondroitin sulfate, and hyaluronic acid. This valuable substance exhibits multiple biological functions such as antitumor activity, immune regulation, fatigue alleviation, and anti-aging effects [2]. Following iron, zinc is the most abundant trace element in humans [3], which represents an essential micronutrient playing crucial roles in numerous physiological processes. These include protein and nucleic acid synthesis, growth and development, immune regulation, wound healing, as well as maintenance of normal taste and appetite [4]. Notably, zinc deficiency has become a widespread nutritional issue affecting all age groups, with a particularly high prevalence among elderly populations [5]. Zinc deficiency has been demonstrated to exert detrimental effects on human physiological functions, particularly manifesting in gastrointestinal disturbances, including impaired digestive function, diarrhea, and anorexia [6]. More severe consequences may include growth retardation, dystocia, and neurological disorders [7]. Recent research advancements have revealed that the formation of zinc–bioactive peptide chelates represents an innovative strategy that not only enhances zinc bioavailability but also confers synergistic biological activities, including but not limited to antioxidant and antimicrobial properties. In the present investigation, we employed low-molecular-weight donkey-hide gelatin peptides (LMW DHGPs) as ligands for zinc chelation, with the scientific premise of integrating the complementary biological advantages of both components to achieve a superior functional performance.

Food-derived peptide–metal chelates exhibit multiple bioactivities, including immunomodulatory, antimicrobial, antioxidant, hypolipidemic, and hypoglycemic effects, along with high bioavailability and rapid absorption [8]. The use of peptide–metal chelates has been extensively investigated as potential agents for the supplementation of essential minerals, including iron, calcium, and zinc. Plant-based peptide sources including wheat [9], rice bran [10], mung bean [11], and walnut [12], as well as animal-derived peptides from Antarctic krill [13], antler plate [14], bovine bone [15], and pork bone [16], have been chelated with calcium or iron to investigate the resulting structures and practical applications. In contrast, research on zinc chelation has primarily involved plant-based materials such as mung bean [17], soybean [18], and coconut cake [19], with limited studies using animal-derived peptide sources. Therefore, this study selected donkey-hide gelatin as a novel peptide source for zinc chelation. On the one hand, donkey-hide gelatin itself possesses significant medicinal value, and investigating donkey-hide gelatin–zinc chelates can promote innovative development and utilization of donkey-hide gelatin-based products. On the other hand, this study establishes a high-yield synthesis process for LMW DHGP–Zn chelates and provides a thorough characterization of their structure and in vitro antioxidant activity, thereby bridging a critical knowledge gap in this field.

First, the optimal preparation conditions for DHGP–Zn chelates have not been systematically established. Parameters such as pH, temperature, and peptide-to-zinc ratio, which are known to critically influence chelation efficiency and the resulting complex’s properties, are largely unreported for this specific system. Second, there is a dearth of detailed structural characterization elucidating the precise coordination mode between DHGP and zinc ions, which is fundamental to understanding its stability and bioactivity. Finally, the antioxidant potential of DHGP–Zn and its correlation with its structure has not been rigorously evaluated across multiple assay systems, leading to a lack of conclusive evidence for its functional benefits. We hypothesized that chelating zinc with LMW DHGP under optimized conditions would yield a stable complex with distinct structural features that confer enhanced antioxidant properties compared to the native peptide. The present study aimed to (1) optimize the preparation process for the LMW DHGP–zinc chelates; (2) comprehensively characterize its structural and physicochemical properties using SEM, UV, FL, FTIR, CD, zeta potential, and particle size analysis; (3) separate it using an ion-exchange column, post-column ninhydrin derivatization, and photometric determination to measure the content and composition of amino acids; and (4) evaluate its in vitro antioxidant activity through DPPH and ABTS radical scavenging assays. This study provides theoretical insights and practical foundations for assessing the bioactivity of LMW DHGP–Zn chelates, expanding the application scope of donkey-hide gelatin, and promoting the development of innovative functional foods. Furthermore, it offers a scientific basis for the potential utilization of LMW DHGP–Zn chelates in the food industry, supporting the advancement of next-generation functional ingredients.

## 2. Materials and Methods

### 2.1. Materials and Reagents

Low-molecular-weight donkey-hide gelatin peptide was purchased from Dong’e Chenkang Pharmaceutical Co., Ltd. (Liaocheng, China) and was not obtained by slaughtering animals. Zinc acetate dihydrate (Zn(CH_3_COO)_2_·2H_2_O, purity ≥ 99.0%) was obtained from Tianjin Damao Chemical Reagent Factory (Tianjin, China). Ethylenediaminetetraacetic acid disodium salt dihydrate (EDTA-2Na, C_10_H_14_N_2_Na_2_O_8_·2H_2_O, purity ≥ 99.0%) and xylenol orange (C_31_H_32_N_2_O_13_S, purity ≥ 85.0%) were obtained from Xilong Scientific Co., Ltd. (Shenzhen, China). Potassium bromide (KBr), potassium persulfate (K_2_S_2_O_8_), formic acid (HCOOH, purity ≥ 98%), 2,2-diphenyl-1-picrylhydrazyl (DPPH, C_18_H_12_N_5_O_6_), and 2,2′-azino-bis (3-ethylbenzothiazoline-6-sulfonic acid) diammonium salt (ABTS, C_18_H_24_N_6_O_6_S_4_) were purchased from Sigma Chemicals Co. (St. Louis, MO, USA). Methanol (CH_4_O), sodium hydroxide (NaOH), hydrochloric acid (HCl), and all other chemical reagents were purchased from Peking Chemical Plant (Beijing, China) and were all of analytical-grade purity.

### 2.2. Optimization of the Preparation Process of LMW DHGP–Zinc Chelates

#### 2.2.1. Preparation of LMW DHGP–Zinc Chelates

The LMW DHGP–zinc chelates were prepared following Chen’s method [20] with modifications. Zinc acetate dihydrate (0.3 g) and LMW DHGP (2.1 g) were dissolved in distilled water (25 mL). The small-molecular-weight donkey-hide gelatin peptides were prepared as previously described [21]. Prior to use, the peptides were desalted using a Sep-Pak C18 cartridge (Waters, Milford, MA, USA). The solution pH was adjusted to 6.0 using 1 M NaOH or 1 M HCl. The mixture was incubated in a water bath at 60 °C for 60 min with continuous stirring. After reaction, anhydrous ethanol (7-fold volume) was added to precipitate the chelates at 25 °C. After standing for 2 h, the chelate precipitate was collected by centrifugation at 4000 rpm for 10 min. The purification of the zinc–peptide chelate was achieved by precipitation with anhydrous ethanol. The mixture was centrifuged, and the supernatant, containing soluble free Zn^2+^ ions, was discarded. The pellet was subsequently washed three times with absolute ethanol to ensure the complete removal of any unchelated zinc. The final purified precipitate was then re-dissolved in deionized water and lyophilized (FD1200-D, FLOM, Qingdao Fulamu Technology Co., Ltd., Qingdao, China) for storage.

#### 2.2.2. Single-Factor Experimental Design

The single-factor experiment was conducted with reference to the method of Xu et al. and modified accordingly [9]. To test the influence of the zinc source concentration on the zinc–peptide chelation rate, zinc salts (0.4–0.8 g) were combined with 7-fold mass equivalents of LMW DHGP, dissolved in 25 mL distilled water, and pH-adjusted to 6.0. The reaction proceeded at 60 °C for 60 min, with subsequent steps following Section 2.2.1.

To test the influence of the peptide–zinc mass ratio on the peptide–zinc chelation rate, zinc salt (0.3 g) was mixed with varying mass ratios (5-9:1) of LMW DHGP, dissolved in 25 mL distilled water, and pH-adjusted to 6.0. The mixture was reacted at 60 °C for 60 min, with subsequent processing following Section 2.2.1.

To test the influence of the chelation pH on the peptide–zinc chelation rate, zinc salt (0.3 g) and LMW DHGP (2.1 g) were dissolved in 25 mL distilled water. The pH was adjusted to values ranging from 4.0 to 8.0, followed by reaction at 60 °C for 60 min. Subsequent procedures followed Section 2.2.1.

To test the influence of the chelation time on the peptide–zinc chelation rate, zinc salt (0.3 g) and LMW DHGP (2.1 g) were dissolved in 25 mL distilled water and adjusted to pH 6.0. The reaction was conducted at 60 °C for varying durations (40, 50, 60, 70, and 80 min), with subsequent steps following Section 2.2.1.

To test the influence of the chelation temperature on the peptide–zinc chelation rate, zinc salt (0.3 g) and LMW DHGP (2.1 g) were dissolved in 25 mL distilled water, pH-adjusted to 6.0, and reacted at temperatures ranging from 40 to 80 °C for 60 min. Subsequent procedures followed Section 2.2.1.

#### 2.2.3. Orthogonal Optimization Experimental Design

Building upon the single-factor experimental results (Section 2.2.2), we systematically evaluated the relative influence of key parameters on the chelation efficiency to optimize the preparation conditions. Three critical factors—zinc source concentration, peptide–zinc mass ratio, and pH value—were selected for an orthogonal test design with an L9 (3^3^) array, with the peptide–zinc chelation rate as the evaluation metric. The corresponding factor levels are presented in Table 1.

In the table above, A1/A2/A3 represent peptide-to-zinc mass ratios of 7:1, 8:1, and 9:1, respectively; B1/B2/B3 denote zinc source concentrations (mg/mL) of 24, 28, and 32, respectively; and C1/C2/C3 indicate pH values of 6.5, 7.0, and 7.5, respectively.

#### 2.2.4. Determination of the Chelation Rate of Peptide–Zinc

The peptide–zinc chelation rate was quantified using EDTA complexometric titration [17]. The reaction solutions obtained under various experimental conditions in Section 2.2.2 were separately processed for analysis. A 5 mL aliquot from each condition was used to prepare the peptide–zinc chelate, which was then diluted to a final volume of 50 mL with pure water. From this diluted solution, a 15 mL portion was transferred to a conical flask, followed by the addition of xylenol orange indicator. The mixture was titrated with a 0.01 mol/L EDTA-2Na solution (dissolved in an acetate–acetic acid buffer, pH 5.5–6.0) until the endpoint was reached. The endpoint was defined as the moment when the color changed from red–violet to a stable bright yellow, and this new color remained stable for 30 s. The volume of EDTA-2Na solution consumed was recorded as V1 (mL). Subsequently, another 5 mL aliquot from each reaction solution in Section 2.2.2 was taken and directly diluted to 50 mL with pure water. A 15 mL portion of this solution was then transferred to a conical flask and titrated in the same manner described above. The volume of EDTA-2Na solution consumed in this titration was recorded as V2 (mL). The peptide–zinc chelation rate (W) was calculated using the following formula:

(1) $$W(\%)=\frac{V1}{V2}\times 100\%$$

### 2.3. Scanning Electron Microscopy (SEM)

For SEM analysis, LMW DHGP and LMW DHGP–zinc chelate powders were mounted on aluminum stubs using double-sided adhesive tape and sputter-coated with gold. Morphological characterization was performed using a scanning electron microscope (Sigma 300, ZEISS, Beijing Prisesta Instrument Co., Ltd., Beijing, China) at magnifications of 100×, 500×, 5000×, and 50,000×, with operational parameters set at 10 mA beam current, 3 kV accelerating voltage, and 13 mm working distance.

### 2.4. Ultraviolet Spectrum (UV)

Determinations were performed using the method of Zhang et al. [22]. For UV-Vis analysis, LMW DHGP and LMW DHGP–zinc chelate powders were dissolved in deionized water to obtain 1 mg/mL solutions. Spectra were acquired from 190 to 400 nm using a UV-Vis spectrophotometer (Multiskan SkyHigh, Thermo Fisher Scientific, Waltham, MA, USA).

### 2.5. Fluorescence Spectra (FL)

Determinations were performed using the method of Wang et al. [23]. The LMW DHGP and LMW DHGP–zinc chelates were, respectively, prepared as solutions with a concentration of 1 mg/mL. The excitation wavelength of the fluorescence spectrophotometer (F97, Shanghai Lingguang Technology Co., Ltd., Shanghai, China) was set at 280 nm, and the emission wavelength range was 300–800 nm. Distilled water was used as the blank.

### 2.6. Zeta Potential and Particle Size Analysis

Determinations were performed using the method of Yuan et al. [24]. Zeta potential and particle size measurements were performed on LMW DHGP and LMW DHGP–zinc chelate solutions (1 mg/mL in water) using a Zetasizer Ultra analyzer (Model Zetasizer Ultra, Malvern Panalytical, Malvern, UK). Samples were filtered through 0.22 µm membranes prior to analysis, with measurements conducted after a 2 min equilibrium period.

### 2.7. Fourier Transform Infrared Spectroscopy (FTIR)

Determinations were performed using the method of Fan et al. [25]. The 2 mg freeze-dried powder of LMW DHGP or LMW DHGP–zinc complexes was mixed with 200 mg dry KBr powder and ground into thin slices, respectively. With KBr as the blank background, the Fourier transform infrared spectrometer (Nicolet iS20, Thermo Fisher Scientific, Waltham, MA, USA) was used for infrared spectrum scanning in the range of 4000–400 cm^−1^ wave number. Each sample was scanned 32 times, and FTIR spectra were obtained. We mixed the LMW DHGP and LMW DHGP–zinc chelates evenly with spectral pure KBr, then formed the tablets and placed them in the optical path. Next, we used an infrared spectrometer to record the spectra of the samples within the range of 400–4000 cm^−1^.

### 2.8. Circular Dichroism (CD)

Determinations were performed using the method of Wu et al. [26]. For circular dichroism analysis, 0.5 mg/mL solutions of LMW DHGP and LMW DHGP–zinc chelates were loaded into quartz cuvettes. CD spectra were acquired from 190 to 270 nm using a circular dichroism spectrometer (Chirascan 100, Applied Photophysics, Leatherhead, UK) at 25 °C. Cuvette path length: 1 cm; protein concentration: 1 mg/mL; spectral bandwidth: 1 nm.

### 2.9. Analysis of Amino Acid Composition and Content

The amino acid composition was detected using an amino acid analyzer (Amino Acid Analyzer, Biochrom 30+, Biochrom Ltd., Cambridge, UK). The freeze-dried samples were hydrolyzed at 110 °C for 24 h with 6 mol/L HCl. The prepared solution was filtered through a 0.45 μm filter membrane and then used for amino acid analysis to obtain the total amino acid data.

### 2.10. Determination of DPPH Free Radical Scavenging Rate

The DPPH radical scavenging activity was assessed according to the method of Liang et al. [21] with slight modifications. Briefly, both LMW DHGP and LMW DHGP–Zn chelate were prepared in a series of concentration gradients (0.1, 0.2, 0.4, 0.6, and 0.8 mg/mL). A DPPH stock solution (0.6 mM) was prepared by dissolving 0.0236 g of DPPH powder in methanol and diluting to a final volume of 100 mL, using a molar extinction coefficient (ε) of 2.40 × 10^3^ L·mol^−1^·cm^−1^ at 517 nm. In a 96-well plate, the sample group consisted of 100 μL of the sample solution, 100 μL of methanol, and 100 μL of the DPPH solution. A blank control (with methanol) was included to correct for the inherent absorbance of the samples themselves. The reaction mixture was incubated at room temperature in the dark for 30 min, after which the absorbance was measured at 517 nm using a microplate reader (JC-1086A, Qingdao Juchuang Environmental Protection Group Co., Ltd., Qingdao, China). The DPPH free radical scavenging rate (%) was calculated by Equation (2), and the antioxidant capacity was ultimately expressed as the IC_50_ value.

(2) $$\mathrm{DPPH}\;\mathrm{free}\;\mathrm{radical}\;\mathrm{scavenging}\;\mathrm{rate}\;(\%)\;=\;\frac{A0-A1}{A0}\times 100\%$$

A0—Absorbance of the blank group;
A1—Absorbance of the sample group.

### 2.11. Determination of ABTS Free Radical Scavenging Rate

The method proposed by Wang [27] was modified. Different concentration gradients (1, 2, 4, 6, 8 mg/mL) of sample solutions were prepared. The ABTS stock solution was prepared by mixing equal volumes of 7 mM ABTS aqueous solution and 2.45 mM K_2_S_2_O_8_ aqueous solution, followed by incubation in the dark at room temperature for 12–16 h. Before use, an appropriate amount of the stock solution was diluted with absolute ethanol to an absorbance of approximately 0.70 ± 0.02 at 734 nm to obtain the ABTS•^+^ working solution. Then, 50 μL of sample solution and 100 μL of ABTS working solution were added to a 96-well plate and thoroughly mixed. After reacting at room temperature for 6 min, the absorbance at 734 nm was measured using a microplate reader. The sample group consisted of 50 μL sample solution and 100 μL ABTS working solution, while the blank control group consisted of 50 μL deionized water and 100 μL ABTS working solution. The ABTS free radical scavenging rate (%) was calculated by Equation (3), and the results were expressed as the IC_50_ value.

(3) $$\mathrm{ABTS}\;\mathrm{free}\;\mathrm{radical}\;\mathrm{scavenging}\;\mathrm{rate}\;(\%)=\frac{A0-A1}{A0}\times 100\%$$

A0—Absorbance of the blank group;
A1—Absorbance of the sample group.

### 2.12. Statistical Analysis

All experiments were performed in at least triplicate (n ≥ 3), and data are presented as the mean ± standard deviation (SD). One-way analysis of variance (ANOVA) followed by Tukey’s post hoc test was used to compare the means among multiple groups. A *p*-value of less than 0.05 (*p* < 0.05) was considered statistically significant. All statistical analyses were conducted using SPSS software (SPSS Inc., Chicago, IL, USA). Graph plotting was performed using OriginPro 2021 (Version 9.8.0.200, OriginLab Corporation, Northampton, MA, USA).

The experimental results were repeated three times. After the data were imported, a significance test was conducted using SPSS 20 software. The significance difference level was set at *p* < 0.05. Graphs were drawn using Origin 2021 software to facilitate the intuitive presentation of the results.

## 3. Results and Discussion

### 3.1. Single-Factor Experimental Results

#### 3.1.1. The Influence of Zinc Source Concentration on the Chelation Rate of Peptide–Zinc

**Figure 1:**
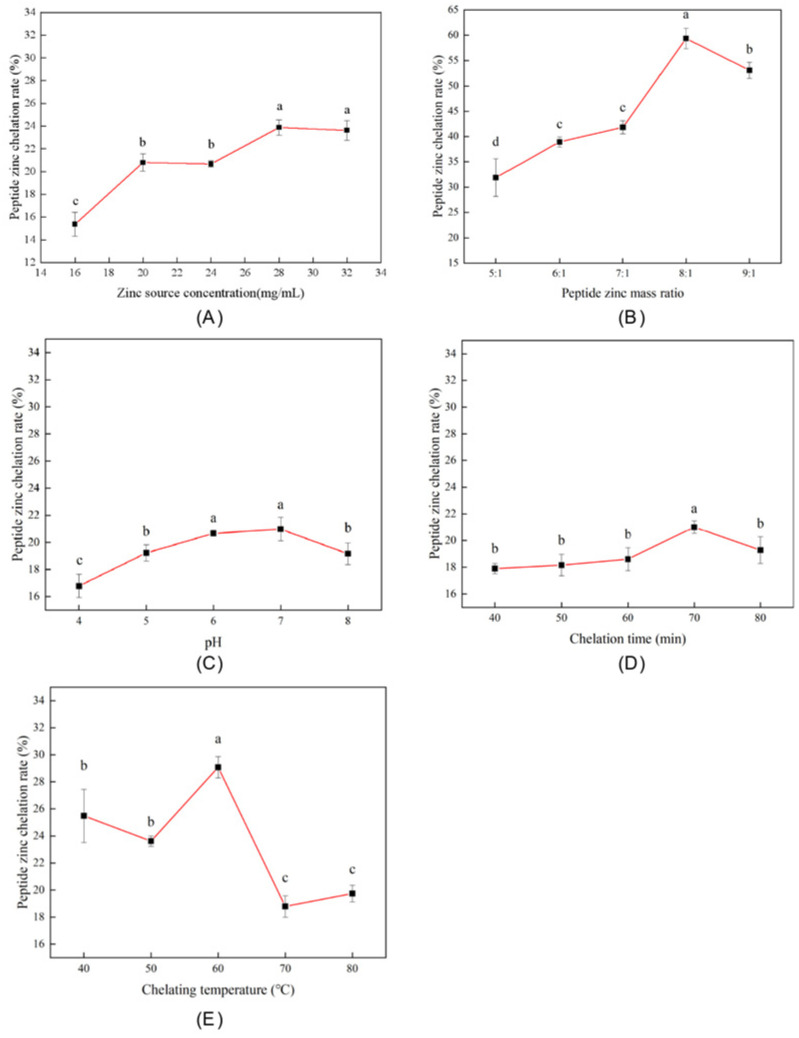
The influence of different factors on the chelation rate of peptide–zinc. (**A**) The influence of zinc source concentration on the chelation rate of peptide–zinc; (**B**) the influence of the mass ratio of peptide–zinc on the chelation rate of peptide–zinc; (**C**) the influence of chelating pH value on the chelating rate of peptide–zinc; (**D**) the influence of chelation time on the zinc chelation rate of peptides; (**E**) the influence of chelation temperature on the chelation rate of peptide–zinc (*p* < 0.05). The same letter (a–d) means that the variance of two samples is not significant (*p* > 0.05), and the different letters (a–d) mean significant (*p* < 0.05).

Figure 1A demonstrates that the LMW DHGP–Zn^2+^ chelation rate generally increased with zinc source concentrations from 16 mg/mL to 28 mg/mL, except for a slight decrease at 24 mg/mL. The maximum chelation rate was achieved at a 28 mg/mL zinc concentration. Beyond this concentration, the solubility of LMW DHGP decreased, reducing the available peptide for chelation and consequently lowering the chelation rate. Thus, 28 mg/mL was identified as the optimal zinc source concentration for LMW DHGP–zinc chelate formation. Similarly, the substrate concentration was selected as a single factor, and the results of calcium on pig bone collagen peptide–calcium chelate, protein peptide–iron chelating agents [26], and rice bran peptide–calcium chelate [10] were also referenced.

#### 3.1.2. The Influence of the Mass Ratio of Peptide–Zinc on the Chelation Rate of Peptide–Zinc

As illustrated in Figure 1B, when the mass ratio of LMW DHGP to zinc salt gradually increases, the chelation rate of the peptide–zinc complex shows a trend of first rising and then decreasing. When the ratio is 1:8, the chelation rate is the highest (59.35%), and if the ratio continues to increase, the rate will decrease. After the reaction between LMW DHGP and zinc ions reaches saturation, the chelation rate of the peptide–zinc complex tends to be the highest. This optimal ratio represents the point at which the available chelating sites (e.g., carboxyl, amino, and imidazole groups) on the peptides are nearly saturated with zinc ions. The subsequent decline in chelation rate beyond this ratio can be attributed to the “screening effect” of excessive positive charges from Zn^2+^ ions, which may neutralize negatively charged binding sites on adjacent peptides, thereby reducing their chelating capacity and potentially causing inter-particle aggregation that precipitates the complex out of solution [12,14,28]. In addition, the chelation rate of different metals with polypeptides is also affected by the characteristics of the metals. For example, the optimal mass ratio of the Agaricus bisporus peptide–calcium chelates [29] is 4:1, and the optimal mass ratio of the wampee seed antioxidant peptide–iron chelate [30] is 3.4:1.

#### 3.1.3. The Influence of Chelating pH on the Chelation Rate of Peptide–Zinc

The pH of the reaction medium was a critical factor, profoundly influencing the chelation rate by modulating the ionic states of both the peptide and the metal ion. As shown in Figure 1C, the chelation rate increased from pH 4.0 to an optimum at pH 7.0 (20.98%), beyond which it decreased. Under highly acidic conditions (low pH), the high concentration of H^+^ ions leads to the protonation of key chelating functional groups (e.g., the carboxylate groups of aspartic/glutamic acid side chains and the imidazole nitrogen of histidine), effectively competing with Zn^2+^ ions for binding sites and suppressing chelation. As the pH approaches neutrality, these groups become deprotonated, enhancing their electron-donating capability and facilitating coordination with zinc ions. The decline in chelation rate under alkaline conditions (pH > 7.0) is likely due to the formation of insoluble zinc hydroxide (Zn(OH)_2_) precipitates, which reduces the concentration of free Zn^2+^ ions available for chelation [3,31]. The diversity in optimal pH values reported in the literature—7.0 for oyster peptides [3], 6.0 for sika deer blood peptides [32], and 8.5 for walnut peptides [31]—can be attributed to differences in the isoelectric points (pI) and the relative abundance of specific chelating residues in different peptide sequences, which alter their protonation equilibria and metal-binding affinity.

#### 3.1.4. The Influence of Chelation Time on the Chelation Rate of Peptide–Zinc

Figure 1D demonstrates that the LMW DHGP–zinc chelation rate increased progressively from 40 to 60 min, showed marked enhancement between 60 and 70 min, then declined from 70 to 80 min. The reaction chelation rate is the best at 70 min (21.01%). The initial increase is driven by the high collision frequency between peptides and zinc ions, promoting the formation of coordination bonds. The observed plateau and subsequent decrease suggest that the reaction reaches a dynamic equilibrium where the rate of chelate formation equals the rate of dissociation. The decline after 70 min could be due to oxidative degradation of the peptide ligands or the slow hydrolysis of zinc ions in aqueous solution, leading to a partial breakdown of the complexes [11,17]. The agreement of this optimal timeframe (60–80 min) with other studies on peptide–zinc chelates [10] confirms that the chelation is a relatively rapid process, governed by the diffusion and binding kinetics specific to the peptide’s molecular structure.

#### 3.1.5. The Influence of Chelation Temperature on the Chelation Rate of Peptide–Zinc

The effect of temperature on the chelation rate was biphasic (Figure 1E). An initial upward trend from 40 °C to 60 °C can be explained by the increased kinetic energy of molecules, enhancing the collision efficiency and accelerating the reaction rate. However, the subsequent decline from 60 °C to 80 °C strongly suggests a thermodynamic limitation and potential structural degradation. Elevated temperatures can provide sufficient energy to disrupt the weak non-covalent interactions (e.g., hydrogen bonding, hydrophobic effects) that stabilize the tertiary structure of peptides, leading to partial unfolding or aggregation. This conformational change can occlude metal-binding sites, thereby reducing the chelating capacity [7,33]. The identification of 60 °C as the optimal temperature aligns with findings for bovine collagen peptide–calcium [15] and pig bone collagen peptide–calcium chelate [16].

### 3.2. The Results of Orthogonal Optimization Experimental Design

Utilizing the peptide–zinc chelation rate as the evaluation index, an orthogonal experimental design was employed to optimize the preparation process parameters for the LMW DHGP–zinc chelate compound. The orthogonal optimization experimental design and its results are presented in Table 2. The results of the orthogonal design experiment were analyzed using range analysis. Factors with larger ranges were found to have a greater impact on the experimental outcomes. Upon analyzing Table 2, it becomes evident that the factors influencing the peptide–zinc chelation rate, in order of significance, are the pH, zinc source concentration, and the peptide–zinc mass ratio. The optimal combination is A3B3C1, meaning the optimal conditions are a 32 mg/mL concentration of zinc acetate dihydrate, a peptide-to-zinc mass ratio of 8:1 (LMW DHGP to zinc acetate dihydrate), a reaction pH of 6.5, a reaction time of 70 min, and a reaction temperature of 60 °C. The experimental results were subsequently verified, and the peptide–zinc chelation rate was determined to be 41.34% ± 0.23%, which was higher than those of other experiments. This confirmed the optimal preparation process parameters.

### 3.3. SEM Analysis

**Figure 2:**
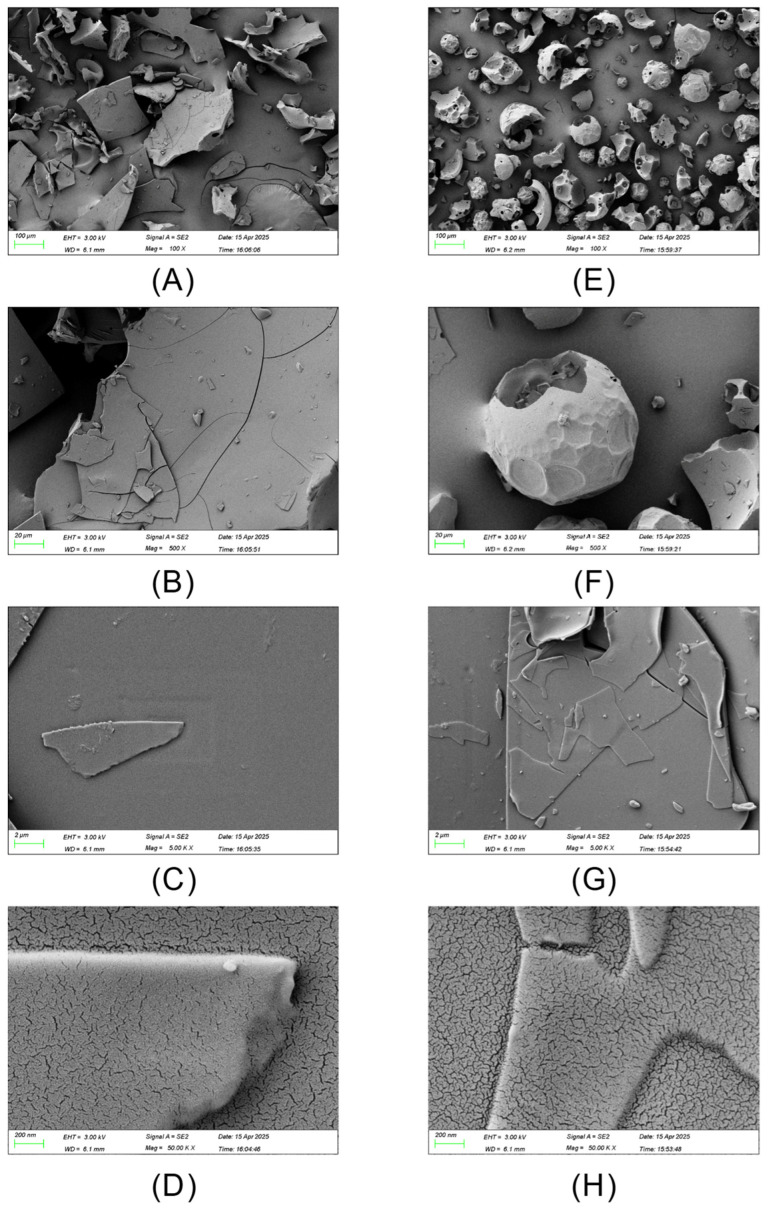
Scanning electron microscopy images of low-molecular-weight donkey-hide gelatin peptides and peptide–zinc chelates. (**A**) LMW DHGP at 100×; (**B**) LMW DHGP at 500×; (**C**) LMW DHGP at 5000×; (**D**) LMW DHGP at 50,000×; (**E**) LMW DHGP–zinc chelates at 100×; (**F**) LMW DHGP–zinc chelates at 500×; (**G**) LMW DHGP–zinc chelates at 5000×; (**H**) LMW DHGP–zinc chelates at 50,000×.

The morphological characteristics of LMW DHGP before and after chelation were observed using SEM electron microscopy, as depicted in Figure 2. Specifically, Figure 2A–D depict the LMW DHGP magnified 100-fold, 500-fold, 5000-fold, and 50,000-fold, respectively; while Figure 2E–H represent the LMW DHGP–zinc chelates at the same magnifications of 100-fold, 500-fold, 5000-fold, and 50,000-fold, respectively. Upon magnification to 100 times, the LMW DHGP exhibits a block-like form, with certain connections between the particles, and the overall appearance is relatively loose. In contrast, the LMW DHGP–zinc chelates reveal a spherical structure composed of various particle sizes and shapes, distributed relatively evenly. Upon magnification at 500 times, the block-like structure of LMW DHGP is clearly visible. The LMW DHGP–zinc chelates predominantly appear as a single particle with an uneven surface; the particles are relatively independent. At magnifications of 5000× and 50,000×, both LMW DHGP and its zinc chelates exhibit a relatively flattened morphology. In summary, the microstructure of the peptides is predominantly blocky, whereas the zinc chelates display fragmented spherical structures, indicating the formation of a new substance. According to studies by Wu [34] and others, the participation of metal ions will accelerate the dimerization reaction of peptides, form dimers, and thus make the structure aggregated and compact.

### 3.4. UV Analysis

**Figure 3:**
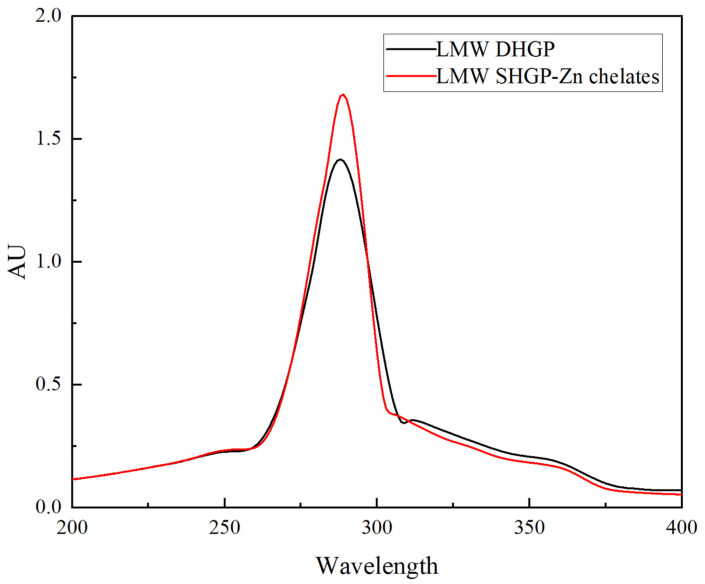
Ultraviolet scanning images of low-molecular-weight donkey-hide gelatin peptides and peptide–zinc chelates.

The differences in the samples following chelation can be analyzed by examining the shifts and intensities of the absorption spectra [35]. As depicted in Figure 3, the maximum absorption peak of the zinc chelate exhibited a notable redshift from 237 nm to 256 nm compared to native LMW DHGP. This significant bathochromic shift indicates the coordination of Zn^2+^ with electron-donating groups on the peptide chain, such as the carboxyl oxygen from the side chains of aspartic acid and glutamic acid, and/or the carbonyl oxygen of the amide backbone. The formation of these coordination bonds stabilizes the energy level of the n-orbital, thereby reducing the energy required for the n-π* electronic transition and confirming the successful formation of a stable peptide–zinc chelate structure [36]. A bathochromic (red) shift was also observed following the chelation of iron with peptides derived from wheat bran globulin hydrolysate [9]. This confirms the occurrence of electronic transitions within the peptide molecules and alterations in some chromophores, leading to the redshift of the absorption peak.

### 3.5. FL Analysis

**Figure 4:**
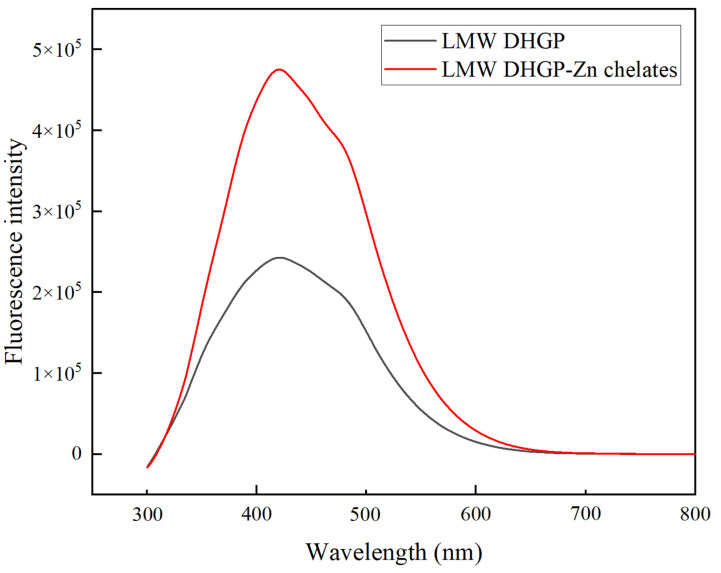
Fluorescence spectra of low-molecular-weight donkey-hide gelatin peptides and peptide–zinc chelates.

The fluorescence spectral changes, specifically a blueshift of the emission maximum from 421 nm to 420 nm and a concurrent increase in intensity (Figure 4), indicate Zn^2+^ chelation by ligand groups within the polypeptide [37,38]. This suggests that zinc binding induces a conformational rearrangement, positioning fluorophores such as phenylalanine and tyrosine in a more constrained and hydrophobic microenvironment. The altered polarity around these residues due to metal coordination further supports the observed spectral shift [39].

### 3.6. Zeta Potential and Particle Size Distribution

**Figure 5:**
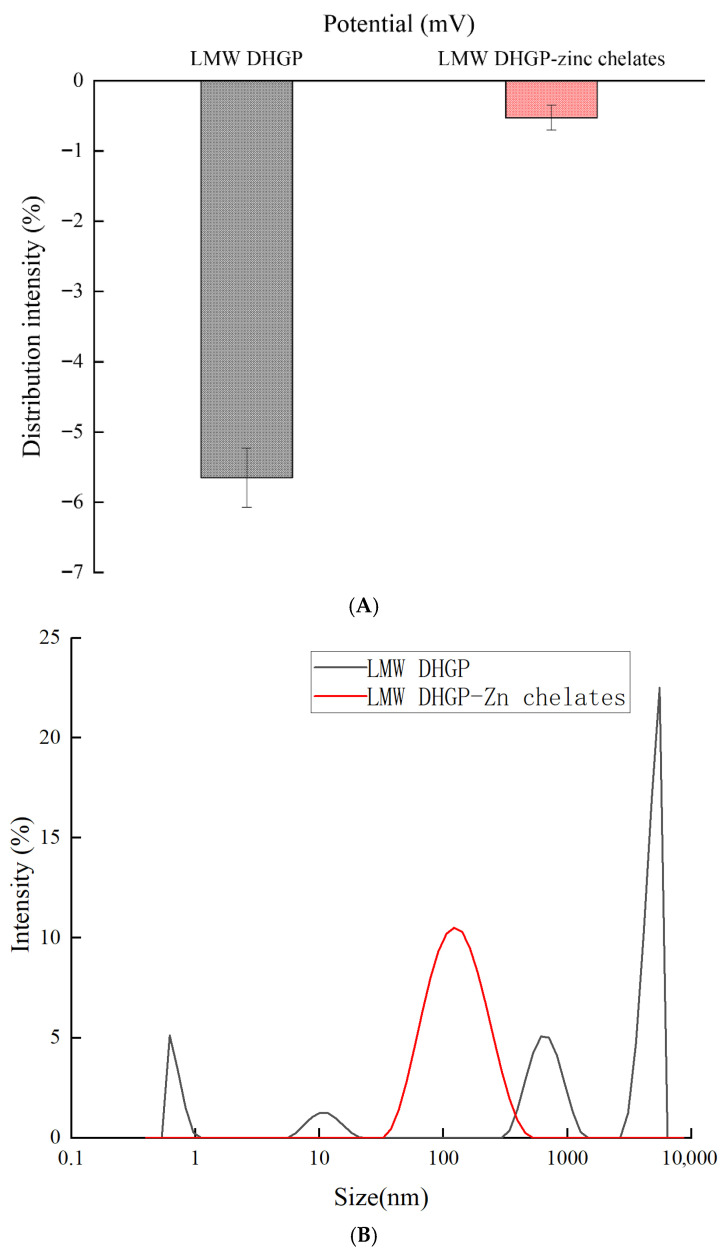
The zeta potential and particle size distribution of low-molecular-weight donkey-hide gelatin peptides and peptide–zinc chelates. (**A**) The zeta potential; (**B**) the particle size distribution.

Significant variations in zeta potential values were observed in LMW DHGP before and after the chelation process. These alterations indicate that chelation substantially modifies both the nature and distribution of surface charges on these molecular structures [40]. The LMW DHGP peptide chain possesses charged groups that confer a net negative electrostatic potential. The introduction of Zn^2+^ likely neutralizes these negative charges on LMW DHGP, thereby altering its surface charge state, as evidenced by the shift in zeta potential from −5.65 mV to −0.525 mV (Figure 5A). This mechanism is consistent with observations in other protein–zinc complexes, such as those derived from soybean meal and walnut peptides [12,41]. Furthermore, the relative content analysis of amino acids suggests that the negatively charged residues, glutamate (Glu) and aspartate (Asp), are key contributors to this potential. We postulate that the carbonyl groups on these acidic amino acid residues in LMW DHGP participate in the reaction with Zn^2+^, analogous to the chelation behavior reported for walnut peptide–zinc complexes [12]. Consequently, it is hypothesized that LMW DHGP primarily chelates Zn^2+^ at these carbonyl sites [42]. Particle size is an important characteristic of metal chelates. The chelation reaction induced a remarkable alteration in particle size distribution (Figure 5B). Prior to chelation, the system was dominated by large particles averaging 5560 nm, with a high polydispersity index (PDI) of 0.528 ± 0.06. This indicates severe aggregation and/or considerable compositional heterogeneity. In contrast, upon chelation with zinc, the particle size was homogenized to a mean of 122 nm, accompanied by a significant decrease in PDI to 0.34 ± 0.05. This demonstrates that the LMW DHGP–Zn complex possesses a more uniform and narrower size distribution. It is noteworthy that the measured size of the peptide solution before chelation far exceeded the pore size of the membrane filter used for sample preparation, even after filtration. This observation further confirms its inherent instability and propensity to aggregate in the absence of zinc, a behavior reminiscent of other aggregating peptide systems like β-casein [43]. In contrast, the chelated product demonstrated superior dispersion stability under the same conditions, a finding consistent with its homogeneous size and lower PDI.

### 3.7. FTIR Analysis

**Figure 6:**
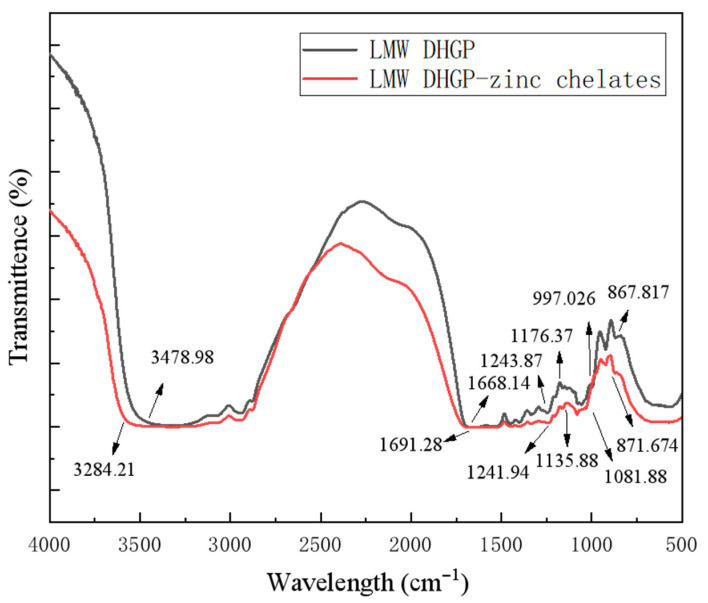
Fourier infrared spectra of chelates of low-molecular-weight donkey-hide gelatin peptides and peptide–zinc chelates.

The Fourier transform infrared (FTIR) spectroscopy analysis provides direct evidence for the coordination between zinc ions and functional groups in the low-molecular-weight donkey-hide gelatin peptide (LMW DHGP) [18]. Comparative spectral analysis revealed characteristic shifts in key absorption bands after chelation (Figure 6). The broad N–H stretching vibration band (3750–3250 cm^−1^) showed significant changes [44] with a specific peak shifting from 2283.32 cm^−1^ to 2387.46 cm^−1^, indicating nitrogen atom involvement in zinc coordination, consistent with findings in β-casein systems [43]. In the amide I region (1700–1600 cm^−1^), the C=O stretching vibration shifted from 1668.14 cm^−1^ to 1691.28 cm^−1^, demonstrating carbonyl oxygen participation in metal binding, similar to observations in iron-chelated scallop mantle peptide [45]. Additionally, the C–O stretching vibration at 997.03 cm^−1^ shifted to 1081.88 cm^−1^, further confirming the role of oxygen-containing groups in coordination, as reported in grisebachin-related studies [44]. These systematic changes across multiple spectral regions collectively demonstrate that both nitrogen atoms (from amino groups) and oxygen atoms (from carboxyl and carbonyl groups) in LMW DHGP served as primary coordination sites for zinc ions, facilitating the formation of stable peptide–zinc chelates, analogous to coordination behaviors observed in zinc-chelated soybean protein hydrolysates [18].

### 3.8. CD Analysis

**Figure 7:**
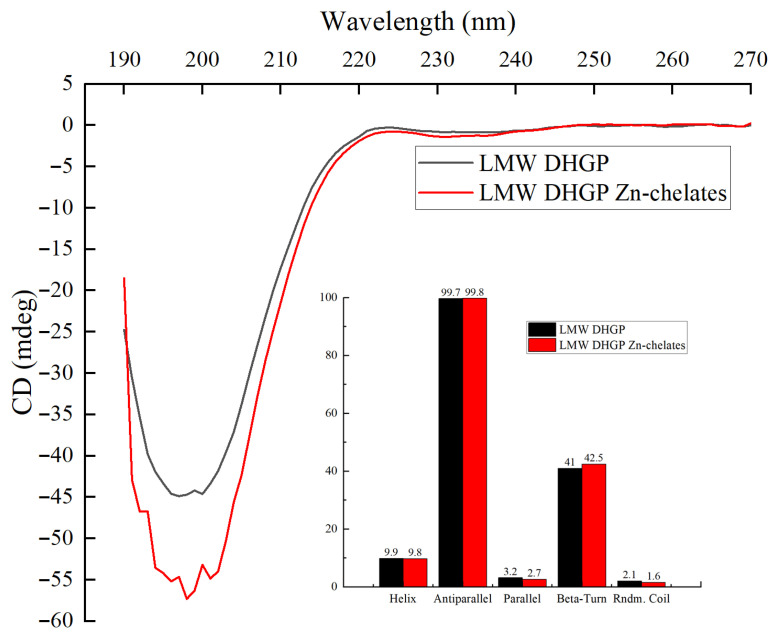
Circular dichroic spectra of low-molecular-weight donkey-hide gelatin peptides and peptide–zinc chelates.

The structure of proteins was studied using circular dichroism spectroscopy [46]. Based on far-ultraviolet circular dichroism (CD) spectral data, the secondary structure of proteins or polypeptides can be elucidated, providing insight into their conformational properties [36]. Therefore, the analysis was primarily conducted in the far-ultraviolet spectral region, below 250 nm. As illustrated in Figure 7, both the LMW DHGP and the LMW DHGP–zinc chelates exhibit negative peaks around 198 nm, with their secondary structure consisting of β-sheets and β-turns, containing a minor proportion of α-helix and random coils. The contents of various secondary structures were obtained using the CDNN 2.1 software. Compared with before chelation, the parallel β-sheet in the peptide–zinc chelates decreased by 0.5%, and the β-turn increased by 1.5%. Although the range of change is small, it further indicates that as the chelates forms, both the secondary structure and the spatial conformation change.

### 3.9. Amino Acid Analysis

The varying affinities of different amino acids for zinc ions primarily stem from structural differences in their side chains and associated charge polarities. Notably, spectroscopic studies have identified carbonyl, carboxyl, and amino groups as exhibiting particularly strong zinc-binding capabilities [38], consistent with their chemical properties as potential coordination sites. Table 3 shows that the amino acid composition changed significantly after zinc chelation: the relative content of acidic amino acids in the low-molecular-weight DHGP zinc chelate increased noticeably (for example, glutamate (Glu) increased from 15.01% to 16.78%, and aspartate (Asp) increased from 7.53% to 8.91%); and the relative content of basic amino acids increased to a lesser extent, indicating they play an auxiliary role in the chelation process. In contrast, the relative content of hydrophobic amino acids generally decreased, such as alanine (Ala) decreasing from 10.62% to 9.88%. According to acid-base theory, the positively charged zinc ion can pair with groups containing oxygen and nitrogen. Therefore, the total amino acid content decreased from 677.940 mg/g to 659.264 mg/g, primarily because the carbonyl groups in aspartate and glutamate participated in the formation of the low-molecular-weight DHGP zinc chelates. In the study of iron chelation by tilapia skin collagen by Ke [47], aspartate and glutamate played important roles in chelation, providing binding potential for the zinc ion.

### 3.10. Analysis of In Vitro Antioxidant Activity

#### 3.10.1. Determination of the Free Radical Scavenging Ability of DPPH

**Figure 8:**
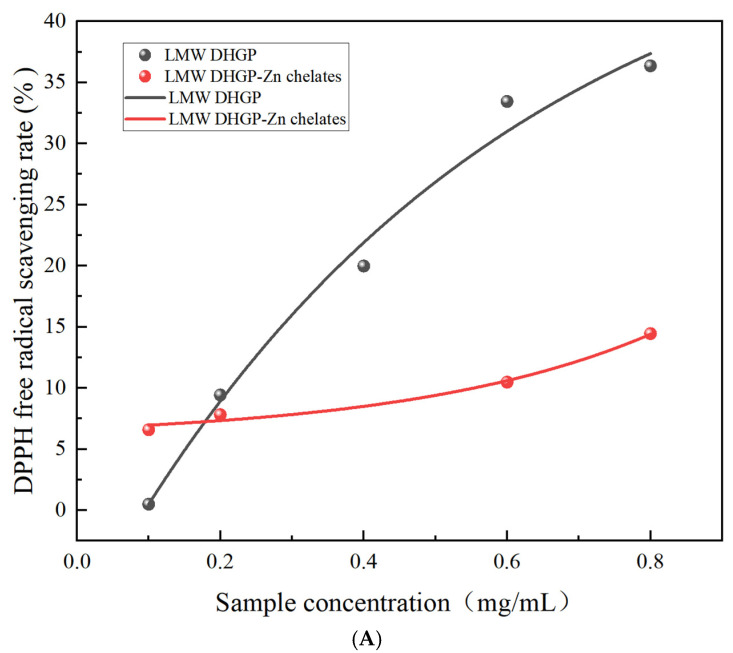

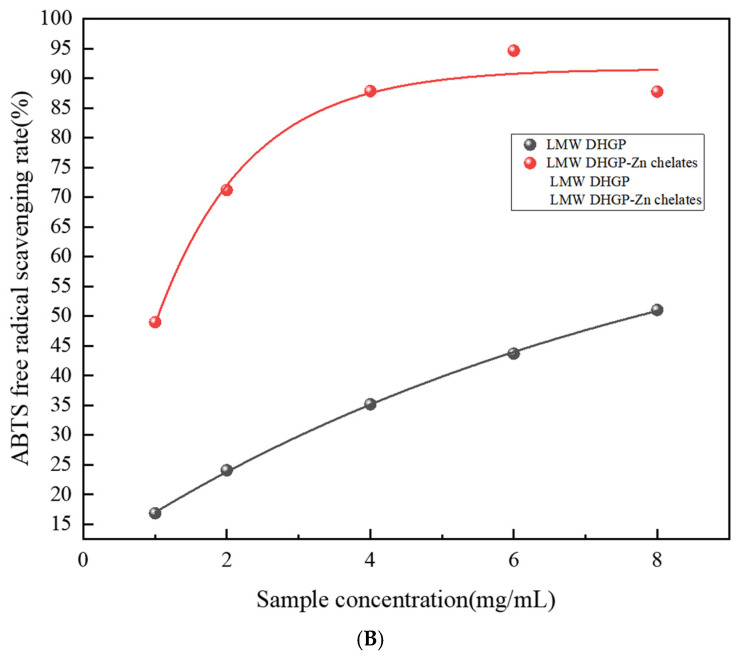
In vitro antioxidant activity of low-molecular-weight donkey-hide gelatin peptides and peptide–zinc chelates. (**A**) The DPPH free radical scavenging ability of low-molecular-weight donkey-hide gelatin peptides and peptide–zinc chelates; (**B**) the ABTS free radical scavenging ability of peptide–zinc chelates and donkey-hide gelatin peptides.

As shown in Figure 8A, the concentrations of LMW DHGP and LMW DHGP–zinc chelate and the DPPH scavenging rate are generally positively correlated. The DPPH scavenging rate of LMW DHGP reaches its maximum at a concentration of 0.8 mg/mL, with a rate of 36.35%; the DPPH scavenging rate of LMW DHGP–zinc chelates reaches its maximum at a concentration of 0.8 mg/mL, with a rate of 14.45%. The DPPH radical scavenging data were fitted using a non-linear regression model (LMW DHGP: y = −61.87 × exp(−(x/0.56)) + 52.30; LMW DHGP–Zn chelates: y = 0.80 × exp(−(x/−0.34)) + 5.90). The LMW DHGP group exhibited an IC_50_ value of 1.85 ± 3.65 mg/mL (R^2^ ≥ 0.95), while the LMW DHGP–Zn chelate showed an IC_50_ of 1.36 ± 0.77 mg/mL (R^2^ ≥ 0.95). These results indicate that the DPPH radical scavenging capacity of the chelate is lower than that of LMW DHGP.

#### 3.10.2. Determination of ABTS Free Radical Scavenging Ability

Similarly, the ABTS radical scavenging activity was analyzed with the same non-linear model (LMW DHGP: y = −66.15*exp(−(x/8.08)) + 75.48; LMW DHGP–Zn chelates: y = −94.03*exp(−(x/1.27)) + 91.59). The scavenging rate increased monotonically with sample concentration, following an exponential saturation growth pattern, which yielded an excellent goodness of fit (R^2^ ≥ 0.95). The LMW DHGP–Zn chelate demonstrated a significantly enhanced antioxidant potency, with an IC_50_ value of 1.04 ± 0.45 mg/mL, which is markedly lower than that of the unchelated LMW DHGP (7.71 ± 1.51 mg/mL).

The higher radical scavenging activity for ABTS of the LMW DHGP–Zn chelate is visually presented in Figure 8B. This enhancement can be attributed to the structural reorganization of LMW DHGP upon chelating zinc ions, which may lead to the exposure of amino acids containing more protons or atomic groups capable of binding with radicals. This observation aligns with the findings of Wu et al. [26], who reported that whey protein peptide–iron chelates possessed significantly higher DPPH scavenging activity than sea cucumber peptides.

It is noteworthy that at excessively high concentrations, potential intermolecular interactions among peptide–zinc chelates might obscure the active sites, thereby reducing the accessibility and reactivity of the chelated molecules with the ABTS radicals and consequently diminishing the scavenging capacity. In contrast to the study by Wang [48], the sample concentration in this work was controlled within the range of 1–10 mg/mL, and the scavenging rate showed a consistent increase with concentration, demonstrating a significant positive correlation for both samples.

## 4. Conclusions

This study used LMW DHGP as the raw material and carried out chelation with zinc acetate dihydrate. The chelation rate of peptides and zinc was used as the index for single-factor experiments and orthogonal optimization experiments, thereby determining the optimal preparation process parameters for the LMW DHGP–zinc chelates: the concentration of zinc acetate dihydrate was 32 mg/mL, the mass ratio of peptides and zinc was 8:1 (LMW DHGP: zinc acetate dihydrate), the chelation pH was 6.5, the chelation time was 70 min, and the chelation temperature was 60 °C. Under these conditions, the chelation rate was 41.34% ± 0.23%. Through SEM, UV, FL, zeta potential, FTIR and CD analysis, the LMW DHGP–zinc chelate presents a spherical structure and is arranged evenly. Absorption peak redshift indicates that zinc ions induce conformational changes in LMW DHGP. Zn^2+^ binds to the polypeptide, altering the microenvironment of the fluorescent groups (residues of Phe and Tyr) on the polypeptide chain. The surface negative charges are neutralized, the potential increases from −5.65 mV to −0.525 mV, the particle size decreases and is uniform (122 nm), and the particles are closely arranged. The main chelation sites are functional groups such as N-H and C=O; the main structure is β-sheet and β-turn. Zinc ions binding to the side-chain groups of acidic amino acid residues are the main reason for induced conformational changes. Compared with LMW DHGP, the LMW DHGP–zinc chelate has a better ability to remove ABTS free radicals, with a removal rate of 94.62%; it also has a certain DPPH free radical removal ability, but it is weakened compared with LMW DHGP. Collectively, the Zn^2+^-induced conformational change in LMW DHGP altered its structure, leading to the formation of a distinct complex. In conclusion, this study successfully optimized the preparation process for donkey-hide gelatin peptide–zinc chelates and demonstrated their favorable in vitro antioxidant activity. The cost advantages derived from the mature local supply chain, combined with the simplicity and scalability of the developed process, underscore the promising industrial potential of these findings. The chelates show potential for development as novel functional products, paving a new path for high-value utilization of donkey-hide gelatin. Future research will focus on investigating their in vivo bioactivity through cell and animal studies, along with a systematic evaluation of digestive stability and toxicity.

## Figures and Tables

**Table 1:** Orthogonal experimental factor table L9 (3^3^).

Horizontal Numbering	Elements		
	A—Peptide–Zinc Mass Ratio	B—Zinc Source Concentration (mg/mL)	C—pH Value
1	7:1	24	6.5
2	8:1	28	7.0
3	9:1	32	7.5

**Table 2:** Orthogonal optimization experimental design results (*p* < 0.05).

No.	Influence Factor			Rate of Chelation (%)
	A	B	C	
1	1	1	1	32.63 ± 0.78
2	1	2	2	31.62 ± 0.62
3	1	3	3	33.52 ± 0.82
4	2	2	1	41.14 ± 1.89
5	2	1	3	32.25 ± 0.48
6	2	3	2	34.73 ± 0.55
7	3	2	3	31.49 ± 0.18
8	3	3	1	41.34 ± 0.23
9	3	1	2	36.83 ± 5.30
K1	32.59	33.90	38.37	
K2	36.04	34.75	34.39	
K3	36.55	36.53	32.42	
Range	3.96	2.63	5.95	
Sequence	C > A > B			
Optimal level	A3	B3	C1	

**Table 3:** Analysis of amino acid composition of low-molecular-weight donkey-hide gelatin peptides and their zinc chelates.

Types of Amino Acids	Relative Content (%)	
	LMW DHGP	LMW DHGP–Zinc Chelates
Asp	7.60	8.98
Glu	15.13	16.92
Gly	23.00	23.51
Ala	10.62	9.88
Val	2.86	2.34
Ile	1.94	1.60
Leu	4.19	3.44
Tyr	1.27	0.92
Phe	2.65	2.11
His	0.86	1.03
Lys	4.58	4.72
Arg	9.75	10.22
Pro	15.41	14.16

Note: The analyses were performed on the purified chelate, Trp undetermined due to hydrolysis.

## Data Availability

The original contributions presented in this study are included in the article, and further inquiries can be directed to the corresponding author.

## Abbreviations

LMW	Low molecular weight
DHGP	Donkey-hide gelatin peptide
SEM	Scanning electron microscopy
CD	Circular dichroism
FTIR	Fourier transform infrared
UV	UV-Vis spectroscopy
DPPH	2,2-Diphenyl-1-picrylhydrazyl
ABTS	2,2-azino-bis(3-ethylbenzothiazoline-6-sulfonic acid) diammonium salt
